# Host Genotype Shapes the Foliar Fungal Microbiome of Balsam Poplar (*Populus balsamifera*)

**DOI:** 10.1371/journal.pone.0053987

**Published:** 2013-01-11

**Authors:** Miklós Bálint, Peter Tiffin, Björn Hallström, Robert B. O’Hara, Matthew S. Olson, Johnathon D. Fankhauser, Meike Piepenbring, Imke Schmitt

**Affiliations:** 1 Biodiversity and Climate Research Centre, Senckenberg Gesellschaft für Naturforschung, Frankfurt am Main, Germany; 2 Molecular Biology Center, Babeş-Bolyai University, Cluj, Romania; 3 Department of Plant Biology, University of Minnesota, St. Paul, Minnesota, United States of America; 4 Department of Biological Sciences, Texas Tech University, Lubbock, Texas, United States of America; 5 Institute of Arctic Biology, University of Alaska Fairbanks, Fairbanks, Alaska, United States of America; 6 Plant Biological Sciences, University of Minnesota, St. Paul, Minnesota, United States of America; 7 Institut für Ökologie, Evolution und Diversität, Goethe Universität Frankfurt, Frankfurt am Main, Germany; Northwestern University, United States of America

## Abstract

Foliar fungal communities of plants are diverse and ubiquitous. In grasses endophytes may increase host fitness; in trees, their ecological roles are poorly understood. We investigated whether the genotype of the host tree influences community structure of foliar fungi. We sampled leaves from genotyped balsam poplars from across the species' range, and applied 454 amplicon sequencing to characterize foliar fungal communities. At the time of the sampling the poplars had been growing in a common garden for two years. We found diverse fungal communities associated with the poplar leaves. Linear discriminant analysis and generalized linear models showed that host genotypes had a structuring effect on the composition of foliar fungal communities. The observed patterns may be explained by a filtering mechanism which allows the trees to selectively recruit fungal strains from the environment. Alternatively, host genotype-specific fungal communities may be present in the tree systemically, and persist in the host even after two clonal reproductions. Both scenarios are consistent with host tree adaptation to specific foliar fungal communities and suggest that there is a functional basis for the strong biotic interaction.

## Introduction

Endophytic fungi live in the tissues of leaves and other plant organs without causing symptoms of disease [1]. Numerous fungi also occur on the surfaces of leaves [2]. Some of the endophytic fungi confer specific traits to their hosts, such as tolerance against heat [3], drought and salinity [4], grazing [5], or pathogen attack [6]. However, for the vast majority of leaf-associated fungi the ecological functions remain poorly known. Early studies of foliar fungal endophytes based on culturing suggested that these communities are hyperdiverse, e.g. [7]. More groups of endophytes were found through the application of environmental PCR [8]. Even greater diversity of fungal endophyte communities was revealed by metabarcoding approaches, thereby providing a more complete inventory of phyllosphere fungi [9]–[11] (by phyllosphere we refer to all fungi associated with leaves, both endophytes and leaf-surface fungi).

Host plant characteristics are known to influence fungal community assembly. It has been shown that plant genotype, taxonomic identity, as well as specific plant traits such as chemical properties can affect microbial community composition and diversity [12]–[17]. For example, host genotype can influence susceptibility to infection with arbuscular mycorrhizal fungi [18] and fungal infection in leaves [19]. Further, transmission mode of the microbial consortia affects fungal community composition. Based on whether the fungi are vertically or horizontally transmitted – and other parameters – Rodriguez et al. [20] classified fungal endophytes into four groups (clavicipitaceous endophytes, class 1: narrow host range, present in grasses; non-clavicipitaceous endophytes, class 2: broad host range, present in diverse plant tissues, low *in planta* diversity; class 3: broad host range, mostly above-ground tissues of plants, horizontal infection of hosts, high *in planta* diversity; class 4: broad host range, present in roots). Vertically transmitted endophytes typically infect their hosts during reproduction, while horizontally transmitted endophytes randomly infect their hosts from environmental sources. The group typically associated with tree leaves is the "class 3", nonclavicipitaceous endophytes. Class 3 endophytes are highly diverse, occur in the above-ground tissues, and are generally considered to be horizontally and stochastically distributed [20]. Despite the prevalence of class 3 endophytes, few studies have demonstrated their ecological functions (but see [6]).

**Figure 1:**
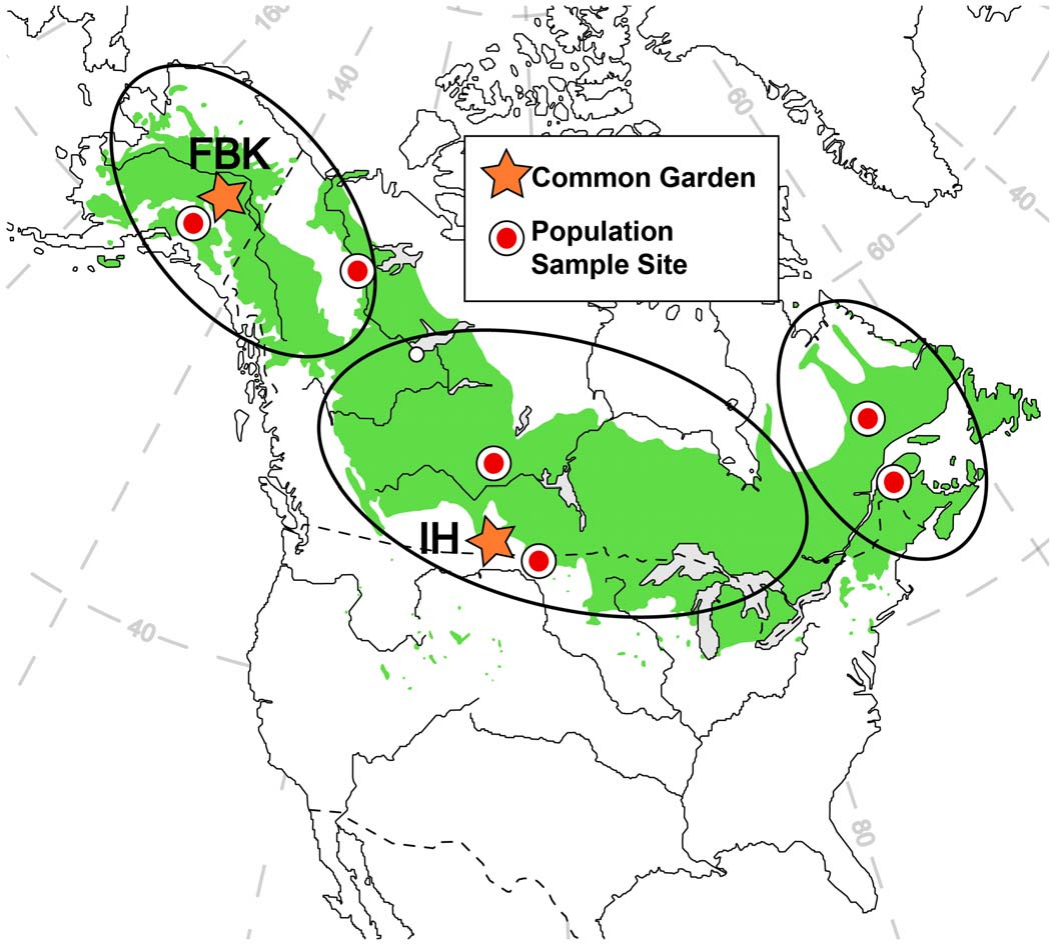
Distribution of balsam poplar. The full natural range of balsam poplar is indicated with green shading [53]. Circles mark the original sampling sites of trees, and stars mark the locations of common gardens (FBK = Fairbanks Garden, IH = Indian Head Garden). The ranges of the three subpopulations identified by Keller *et al.* [21] are indicated with large ellipses.

In this study we investigate potential effects of the host genotype in shaping the composition of the foliar fungal communities of balsam poplar (*Populus balsamifera* L.). This is a well-studied North-American tree species, with a vast range covering most of Canada, Alaska, and the northern U.S.A. The current distribution is the result of a northward range expansion after the last glacial maximum [21]. Recent studies revealed evidence of population structure, with three regional subpopulations, each characteristic of a major geographic area of the tree’s range (Fig. 1, [21]), as well as extensive local adaptation [21], [22]. In order to determine if plant genotypes structure the foliar microbiome of balsam poplar, we investigated whether trees belonging to different regional subpopulations (genotype groups) that were planted into a common garden have specific leaf-associated fungal communities. To accomplish this goal we used 454 amplicon sequencing of fungal ITS sequences from DNA extracted from poplar leaves. We found that the genotype of the host tree structured its foliar fungal community.

## Materials and Methods

### Ethics Statement

No specific permits were required for the described field studies. The study site is not protected in any way, and the study did not involve endangered or protected species.

### Experimental Setup

In 2010, balsam poplar leaves were sampled from 23 trees growing in a common garden near the northern edge of the species range in Fairbanks, Alaska. These trees originated from cuttings that were originally sampled from six geographically defined populations during the winter of 2005–2006. The original cuttings were rooted and grown in a common garden at the Canadian Agroforestry Development Centre in Indian Head (Saskatchewan, Canada) since 2007. In 2009 cuttings from these trees were sent to Fairbanks, Alaska, rooted in the greenhouse, and planted into a new common garden. The newly rooted trees originating from these cuttings grew for 12 months in the Fairbanks common garden until our sampling in 2010. The Fairbanks garden is located on a cleared area of the University of Alaska campus (64.87°N, 147.86°W). The opening is surrounded by coniferous forest. All trees were genotyped using single nucleotide polymorphisms (SNP) from 590 gene fragments [23]. Based on 423 SNPs derived from these regions Keller et al. [21] found a strong phylogeographic pattern in balsam poplar (Fig. 1). Keller et al. were able to distinguish three significantly different, geographically confined subpopulations of *P. balsamifera*. In our study the “northern” and “central” subpopulations were represented by eight genotyped trees each, and the “eastern” subpopulation was represented by seven genotyped trees. At the time of sampling, the trees were approximately 25 cm high, with only a few leaves. We collected one healthy, similarly sized leaf per specimen. We sampled only one leaf per tree to avoid damaging the saplings. According to Cordier et al. [24], the similarity of fungal assemblages increases with decreasing distance within the same tree canopy. Thus, we assumed that there are no great differences among phyllosphere fungi of the same sapling, given the small size of the trees. Although foliar fungal communities may differ among the leaves of host individuals [24], we assumed that the number of host specimens per genotype group (7–8) is sufficient to account for the uncertainties concerning intra-host diversity.

### DNA Procedures and Sequencing

To ensure that the fungal communities did not change during transportation to the lab we rapidly dried the leaves by placing them immediately in silica gel. Leaves dry very rapidly under these conditions (within a few hours from the time of the sampling). We assumed that the rapid drying also prevents preferential fungal growth in the collected leaves (i.e., the growth of fungal strains that cope better with drying conditions). Once dried, the sampled leaves were carried to the lab in plastic bags filled with silica gel. Within 2 months of sampling, DNA was extracted from dried leaves using Qiagen DNeasy Plant Mini Kit (Qiagen Inc. USA), without surface-sterilization. We are aware that avoiding surface-sterilization may cause further complexity in the data. We consider that our dataset represents both endophytic communities and fungi found on the surfaces of the leaves.

DNA extraction, PCR conditions, and primers can strongly influence community composition recovered by amplicon sequencing [25], [26]. For this reason, we treated all samples simultaneously and identically in order to minimize biasing our representation of the fungal community.

Using ITS1F (CTTGGTCATTTAGAGGAAGTAA) and ITS4 (TCCTCCGCTTATTGATATGC) fungal primers [27], [28] for PCR we amplified the entire ITS region with TaKaRa ExTaq polymerase (Clontech Laboratories, Inc. USA). We pooled three PCR replicates with different annealing temperatures (52°C, 55°C, 57°C). PCR amplifications were run for 35 cycles. To allow multiplexing of PCR products during the 454 sequencing we labeled gel-purified PCR products (QIAquick Gel Extraction Kit, Qiagen, Inc. USA) in a short tagging PCR with MID-labeled fusion primers (template specific primers+MID tags +454 key +454 adapter sequences, Table S1) for 6 cycles. We decided to use this second, short PCR for sample tagging because the 35 cycle PCR reactions with fusion primers provided unreliable results (data not shown).

Tagged PCR products were also gel-purified. This was followed by an extra cleaning with SPRI beads (Agencourt AMPure XP, Beckman Coulter, Inc. USA). We quantified the tagged PCR product concentration with Quant-iT™ PicoGreen®dsDNA assays (Invitrogen, Inc. USA). MID-tagged community amplicons from five additional, non-genotyped poplar specimens were also multiplexed in the same sequencing run, along with the PCR products for the current study [29]. The overall number of multiplexed samples was 28. Product concentrations were then normalized, and products were sequenced on two 1/4th and two 1/16th plate fractions on a Roche/GS FLX+ platform with Titanium chemistry by the High-Throughput Sequencing and Genotyping Unit of the Roy J. Carver Biotechnology Center in Urbana, Illinois. We followed the recommendations in Nilsson et al. [30] for a standardized characterization of our next-generation fungal community dataset. Raw sequence data were deposited in the European Nucleotide Archive (ENA) as ERP001860.

### De-noising of 454 Sequences

After removing MID tags, binary SFF files were converted into text flowgrams with sffinfo v2.5.3, a program from the 454 Sequencing System software (454 Life Sciences). Trimming information provided by the sequencer in the raw data files was used for a preliminary trimming of the 3′ ends of poor quality fragments of the flowgrams with sffinfo. The trimmed flowgrams were processed with the AmpliconNoise v1.23 pipeline [31] (min. flowgram length: 360; max. flowgram length: 720; PyroNoise cluster size *s* = 60, PyroNoise initial clustering cutoff *c* = 0.01; SeqNoise cluster size *s* = 30, SeqNoise initial clustering cutoff *c* = 0.08). Trimmed reads shorter than 300 bp were discarded, and all reads were truncated to 450 bp to remove noisy ends. Chimera checking was performed with PerseusD [31]. Pruned sequences produced by the 454 runs for the foliar fungal communities of each balsam poplar host specimen are provided in FASTA format (Material S1). For downstream analyses we retained only 5′–3′ oriented forward reads containing the perfectly matched 5′–3′ forward primer. In most cases these fragments contain a small part of the nuclear small ribosomal subunit (18S), adjacent to the primer, ITS1, 5.8S, and ITS2. Because conserved DNA fragments may result in erroneous taxonomic assignment of sequences during BLAST searches [32], we removed the fragment coding for the ribosomal small subunit (SSU) and 5.8S and kept only complete ITS1 and incomplete ITS2 regions from the cleaned 454 reads for subsequent BLAST searches. These were identified with the FungalITSExtractor utility [32].

### BLAST and Taxonomic Assignment in MEGAN

We downloaded all annotated fungal ITS1, 5.8S, and ITS2 sequences from NCBI (Feb. 24, 2010) and used these to build a BLAST v2.2.21 [33] database against which we blasted both ITS1 and ITS2 fragments. We assigned sequences to the lowest common ancestor (LCA) of known organisms using MEGAN v4 [34]. If a read matches several database sequences with similarly high scores, then it is assigned to the lowest common phylogenetic ancestor of these reads. Some of the GenBank reads might be problematic, but the poorly annotated reads will only shift the assignment toward the safety of LCA (generally at lower taxonomic resolutions). Our settings for the LCA algorithm were: minimum number of reads (Min Support): 1, minimum BLAST bit score (Min Score): 200, bit score percentage of all considered BLAST hits sequences compared to the highest score (Top Percentage): 5. We tried to use both extracted ITS1 and ITS2 fragments as paired-end data during the MEGAN assignment, but because of their short length, none of the ITS2 fragments passed the bit score filter. Consequently, all taxonomic assignments are based on the extracted ITS1 reads. The number of pruned 454 reads assignable by MEGAN to low-level taxa (125) are available in Material S2. Although we acknowledge the issues that may arise from the use of GenBank sequences (e.g. the annotation of the sequences not always being complete or trustworthy) [34], we think that our sequence data treatment, and the taxonomic assignment by parsing BLAST results in MEGAN accounts for most of the issues arising from poorly annotated sequences and chimeras which might be present in the GenBank.

### Rarefaction Analysis

We removed all sequences not identified as being of fungal origin by the previous BLAST/MEGAN assignment. For the rarefaction analysis we clustered the remaining forward reads (including the SSU fragment, the ITS1, the 5.8S, and a fragment of the ITS2) with a grammar-based approach in GramCluster v1.3 [35]. In this approach an alphabet is defined as a set of finite, nonempty symbols. These symbols form finite-length sequences, or strings. A language is considered as a subset of strings, which are selected from all strings over an alphabet. The question is whether a string is a member of some particular language. As languages might be infinite, a compact description of the strings in a given language is defined as a grammar. In this approach, the “gramma” of the new sequences is compared with cluster-representative sequences. If a sequence does not fit into a suitable cluster, a new cluster is established. We used 6 grammar-based clustering thresholds (0.17, 0.15, 0.13, 0.11, 0.1, 0.09). These thresholds roughly correspond to 80%, 85%, 90%, 95%, 97% and 99% sequence similarities. Russell et al. [35] compared sequence similarities of a generated sequence set at 95%, 90%, 85% similarity thresholds with grammar-based clustering thresholds 0.11, 0.13, 0.15. We interpolated the other three thresholds assuming a linear relationship. Rarefaction was calculated for reads pooled for all samples in Mothur v1.21.1 [36]. Clusters delimited at these 6 grammatical threshold levels with GramCluster are available in Material S3. We also clustered forward sequences that were quality-trimmed, but not processed with AmpliconNoise at 97% threshold to evaluate the effects of the data pruning step on the recovered cluster numbers.

### Analysis of Community Structure

We analyzed fungal community structures by selecting all BLAST hits assignable to the lowest taxonomic level (called "leaves" in MEGAN). We completed this matrix with all hits assigned to the genus level. This resulted in a taxonomic matrix consisting almost exclusively of species and genera, containing also six families (Material S2). We omitted all other reads assignable only to higher taxonomic levels. We checked the saturation of sampling with species accumulation curves in R [37], using the package vegan v2.0–2 [38]. We estimated the likelihood that our taxa list fits a Poisson lognormal distribution [39] with the R package poilog v0.4 [40], with 10,000 bootstrap replicates. In case of a good fit this allows estimating the fraction of taxa recovered by the sampling.

We used linear discriminant analysis (LDA) as implemented in the R package MASS [41] to test the hypothesis about the effects of the three plant genotype groups identified by Keller et al. [21] on the basis of 423 single nucleotide polymorphisms. Taxa encountered only once during the taxonomic assignment and clustering steps were considered accidental and discarded. Taxa found in fewer than four host trees in the entire dataset were also considered accidental and removed. Based on their closest BLAST hits we attempted to assign potential ecological functions to discriminating taxa. We complemented the LDA with a general linear model-based (GLM) analysis, as implemented in the R package mvabund [42]. GLM tools are able to deal with signals of different sampling depths resulting from a constant sampling effort; mvabund uses a resampling-based hypothesis testing to reveal factors associated with structures in multivariate abundance data. We tested our hypothesis about the host genotype effect on the foliar fungal community composition by computing an analysis of deviance for multivariate generalized linear model fits using likelihood-ratio-tests and Monte Carlo resampling with 999 iterations.

## Results

The 454 Titanium runs on two 1/4th plate, and two 1/16th plate fractions produced 204,052 reads. Of these reads 203,459 featured correct MID tags (corresponding to the 28 samples multiplexed for sequencing). After quality trimming of read ends, filtering for overall sequence quality and chimeras, and discarding short (less than 300 bp) reads, 126,402 reads were retained (between 3,315 and 7,603 reads from each of the sampled trees). The final number of reads after filtering for 5′-3′ oriented forward sequences was 51,596. Individual samples contained 1,388–3,057 forward-oriented reads.

**Figure 2:**
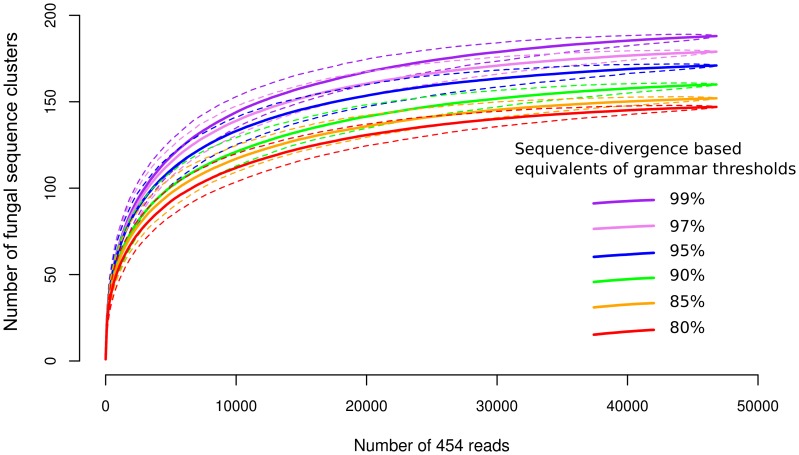
Rarefaction curves of pooled 454 reads at 6 grammar thresholds. Sequence-divergence based equivalents of grammar thresholds are shown in the figure. Dashed lines show 95% highest and lowest confidence intervals of rarefaction curves.

The combined BLAST/MEGAN analysis assigned the reads to 125 taxa. The communities were dominated by a few very abundant taxa. However, most of the taxa were rare, 80% of them being represented by less than 43 reads combined in the 23 host clone leaves (Table 1). Individual trees had 11–44 assignable taxa (Material S2). Grammar-based clustering of fungal reads at a threshold equivalent to 97% sequence similarity delimited 179 fungal sequence clusters (Fig. 2) for the entire dataset. Lists of these clusters are provided in Material S3, for all 6 sequence similarity threshold levels (80%, 85%, 90%, 95%, 97%, 99%), along with their representative sequences. We obtained 621 sequence clusters at 97% clustering threshold after clustering quality-trimmed, but not AmpliconNoise-processed sequences.

**Table 1:** The 25 most common fungal taxa assigned on the basis of ITS1 reads to foliar fungal communities of balsam poplar.

		Division	Order	Lifestyle	GenBank Taxonomy ID	Sum of reads	Most reads/genotype	References
*Leptosphaerulina* sp.	*	Ascomycota	Pleosporales	plant parasitic, endophytic	55172	4913	839	[54], [55]
*Ampelomyces quisqualis*	*	Ascomycota	Pleosporales	hyperparasitic on Erysiphales, saprotrophic, endophytic	50730	1199	258	[56], [57]
*Sclerostagonospora* sp.	*	Ascomycota	Pleosporales	saprotrophic, endophytic	759790	753	145	[58]
*Cryptococcus* sp.	*	Basidiomycota	Filobasidiales	saprotrophic yeast, pathogenic in humans, endophytic	107441	746	126	[56], [59]
*Podosphaera* sp.	*	Ascomycota	Erysiphales	plant parasitic (host specific)	62701	547	96	[54]
*Cryptococcus* sp.	*	Basidiomycota	Tremellales	saprotrophic yeast, pathogenic in humans, endophytic	106841	520	99	[56], [59]
*Blumeria graminis*	*	Ascomycota	Erysiphales	plant parasitic (host specific)	34373	335	53	[54]
*Periconiella* sp.	*	Ascomycota	Capnodiales	plant parasitic, saprotrophic, endophytic	487386	319	87	[60]
*Ascochyta hordei* var. *hordei*	*	Ascomycota	Pleosporales	plant parasitic, endophytic	565415	296	115	[61]
*Sporobolomyces* sp.	**	Basidiomycota	Sporidiobolales	saprotrophic yeast	243824	259	81	[54]
*Minimidochium* sp.	*	Ascomycota	incertae sedis (Pezizomycotina)	saprotrophic	648993	216	44	[62]
Sclerotiniaceae *sp.*	*	Ascomycota	Helotiales	plant parasitic, saprotrophic	28983	156	47	[54]
*Articulospora proliferata*	*	Ascomycota	Helotiales	aquatic hyphomycete, endophytic in roots	556895	145	26	[63]
*Cryptococcus macerans*	*	Basidiomycota	Tremellales	saprotrophic yeast, endophytic	89926	119	31	[56], [64]
*Bullera globospora*	*	Basidiomycota	Tremellales	saprotrophic yeast, endophyte	4976	104	22	[61]
*Phaeosphaeria* sp.	*	Ascomycota	Pleosporales	saprotrophic, associated to leaves	55067	103	17	[59], [61]
*Oculimacula yallundae*	*	Ascomycota	Helotiales	saprotrophic, plant parasitic	86028	86	28	[65]
*Cryptococcus oeirensis*	*	Basidiomycota	Tremellales	saprotrophic yeast	104410	57	12	[56]
*Rhodosporidium lusitaniae*	*	Basidiomycota	Sporidiobolales	saprotrophic yeast	33191	57	13	[66]
*Cryptococcus paraflavus*	*	Basidiomycota	Tremellales	saprotrophic yeast, endophytic in grasses	257870	53	9	[59]
*Leucosporidium golubevii*	*	Basidiomycota	Leucosporidiales	saprotrophic yeast	231204	48	7	[67]
*Fusicladium* sp.	**	Ascomycota	Pleosporales	plant parasitic, endophytic	50375	44	14	[68], [69]
*Pyrenophora tritici-repentis*	*	Ascomycota	Pleosporales	plant parasitic, saprotrophic	45151	44	12	[56]
*Exophiala* sp.	*	Ascomycota	Chaetothyriales	saprotrophic, endophytic	470462	44	17	[56], [70]
*Chalara* sp.	*	Ascomycota	Microascales	plant parasitic, endophytic	220407	43	17	[56], [61]

* taxa discriminating among host specimen genotype groups in the linear discriminant analysis;

** taxa with above-average discriminating power.

**Figure 3:**
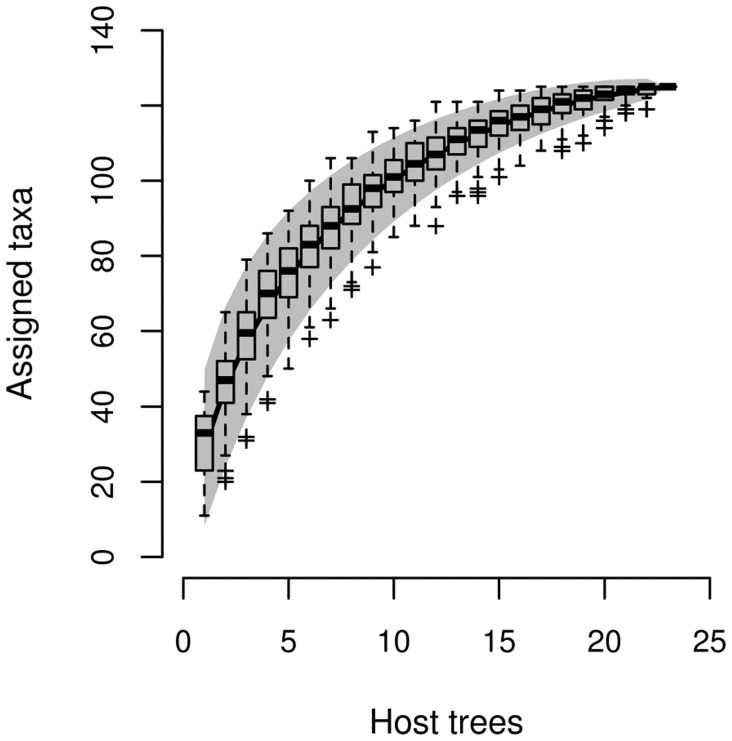
Species accumulation curve of assigned taxa. Boxplots mark standard deviations. Gray shading represents confidence intervals.

We plotted different similarity thresholds and showed that sequence cluster discovery approaches, but does not reach, a saturation plateau (Fig. 2). The species accumulation curve also approaches a saturation plateau (Fig. 3). Fitting the observation data to Poisson lognormal distribution suggests that we sampled approximately 83% of the assignable taxa (goodness of fit to Poisson lognormal distribution *gof* = 0.475).

**Figure 4:**
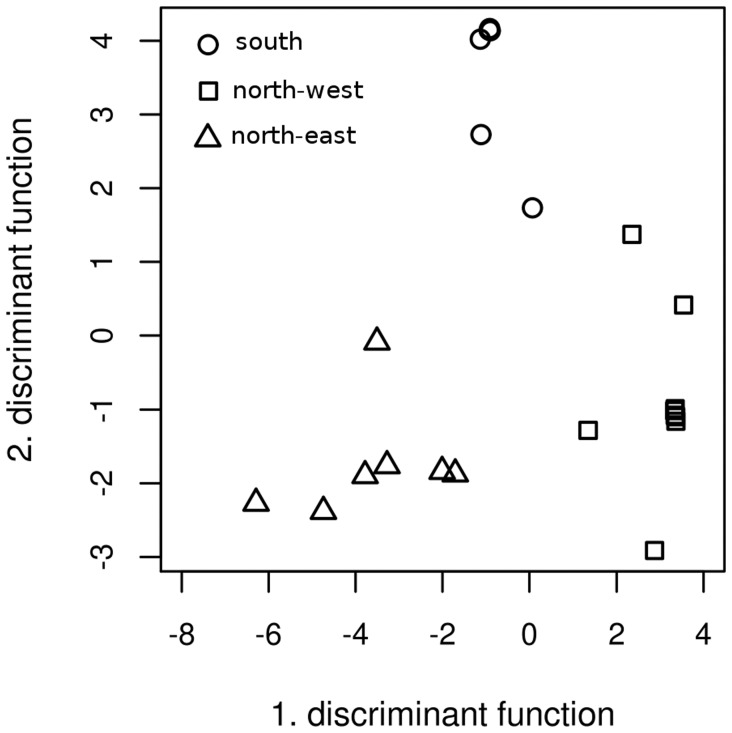
Linear discriminant analysis of fungal communities. *A priori* grouping of the LDA is based on host tree genotypes. Symbols on the plot represent the genotype group of the tree.

Linear discriminant analyses revealed that the genotype of the poplar trees and the composition of their foliar fungal community are tightly linked (Fig. 4). Most foliar fungal communities significantly grouped into the *a priori* groups defined on the basis of their host genotypes (Table S1). Of the 125 taxa assignable in MEGAN, 55 were found to be strongly discriminating (Table S2). The strongly discriminating taxa had similarities to species known to be saprotrophs (41), endophytes (31), plant (23), and animal pathogens (6). The analysis of deviance of multivariate GLM fits confirmed the results of the LDA analysis. The GLM results also show strong genotype effects on foliar fungal community composition (residual degree of freedom *rdf* = 20, degree of freedom *df* = 2, deviance *D* = 3767, *p* = 0.001).

## Discussion

Our survey of the leaf-associated fungal communities found in and on poplar leaves sampled from a common garden revealed that the host genotype is a significant determinant of the composition of leaf-associated fungal communities. This finding is in conflict with the expectation that foliar fungal communities represent a random sample of fungi present in the environment. The results imply the existence of specific relationships between the host tree and its foliar fungal microbiome. Intriguingly, the geographic divergence of the balsam poplar subpopulations happened relatively recently, after the last glacial maximum (∼18,000 years ago [21]). This suggests that host-phyllosphere fungal relationships may form within relatively short geological timeframes.

There are only few previous studies showing that closely related host plant species (e.g. [43], and sometimes different genotypes of the same species [12], [19], [24] are associated with characteristic leaf endophytic fungi. Our results are consistent with these findings that the composition of phyllosphere fungal communities may be partly determined by the genetic makeup of their host (Fig. 4A,B). The results suggest that the hypothesis of completely random, horizontal infection of trees by leaf-associated fungi can be rejected. The trees seem to have unique fungal communities, even after two clonal translocations and two vegetation periods spent in common gardens.

Taxa discriminating among genotype groups in the analyses have close affinities to species with diverse ecological functions (Table S2), including saprotrophs (41/55), endophytes (31/55), and plant (23/55) and animal parasites (6/55). Surprisingly we found the lichen-forming genus *Usnea* among the discriminating taxa. We think it is likely that we sequenced epiphyllous diaspores. This shows that due to the relatively low sample numbers in our study, the possibility of picking up signal from contaminant taxa cannot be completely ruled out. We expect that increasing the number of samples can overcome this problem. An effective removal of all DNA from the leaf surfaces may serve the same purpose, although we had doubts about the potential uniformity of sterilization of uneven balsam poplar leaves. Other discriminating taxa (Table S2) include species found in other Salicaceae (*Crocicreas culmicola*), relatives of known plant parasites (*Mycosphaerella* sp.). Some reads were assigned to unexpected taxa, *e.g. Blumeria graminis* and *Pyrenophora tritici-repentis* (both known to occur only in Poaceae), *Leucosporidium golubevii* (known from freshwaters). The unexpected assignments may also result from epiphyllous diaspores. Alternatively, the unexpected hits may come from presently unknown taxa, which are happen to be closely related to species present in GenBank. The list of BLAST hits should also be interpreted cautiously, as it strongly relies on the quality of our reference database (fungal sequences in GenBank, [44]). Although we assume that the MEGAN-based taxonomic assignment deals with many problems arising from poor GenBank entries, well annotated phyllosphere fungal sequences are likely to be underrepresented in any public database because of methodological difficulties in their study. There are many uncertainties about the taxonomy of even relatively well-studied species (e.g. plant parasitic microfungi, [45]).

Each of the 25 most common taxa had some discriminating value in the LDA, but only two of them had above-average discriminating values (Table 1). *Sporobolomyces* spp. (259 reads) are known as ballistosporic yeasts without host specificity. The genus *Fusicladium* (corresponding to the asexual forms of *Venturia* spp.) includes almost 100 species, some of them known to be highly host specific, as shown for example on *Populus* spp. by Newcombe [46]. Each of the other 16 strongly discriminating taxa were represented by less than 43 reads in all 23 host leaves combined. This suggests that many of the genotype-specific taxa may be among the relatively rare members of the foliar fungal communities in balsam poplar. The rarity of the discriminating taxa also provides a plausible explanation for the scarcity of reports on the host-specificity of foliar fungal tree endophytes. If the relevant taxa are relatively rare, only high resolution next-generation metabarcoding (*sensu* Pompanon et al. [47]) studies may reliably reveal specific host-foilar fungal relationships.

Similar to previous reports on tree leaf endophytes characterized by 454 pyrosequencing [9], [10] we found highly diverse leaf fungal communities on balsam poplar. However, the communities reported here seem far less diverse (Fig. 2) than those reported by Jumpponen & Jones [9]: they recovered about 700 phyllosphere fungi from ∼18,000 pyrosequencing reads. The lower diversity we estimate may be a biological fact resulting from differences between the two host species. Sampling strategy, the age and size of the leaves, host trees, and the higher latitude of the common garden may also affect the number of the recovered taxa. However, we find it unlikely that the sampling itself is responsible for the lower diversity we report here, as 1) similar to us, Jumpponen & Jones [9] also sampled one leaf/tree, 2) in case of vertically transmitted fungi the young trees should directly obtain their phyllosphere fungi from older plants, 3) during yearly horizontal infections from aerial spores we expect similar numbers of fungi infecting the leaves of young and old trees. The latitude of the common garden may play an important role in the observed differences, as it was shown that boreal endophyte communities are less species-rich compared to temperate and tropical communities [48]. There are several aspects of data processing that may considerably affect the estimated phyllosphere fungal community. In particular, we applied denoising and chimera-removal methods [31] recently developed for error types apparent in 454 amplicon sequencing (homopolymers, chimeric PCR artifacts). De-noising next-generation sequencing datasets is especially important in the case of highly diverse communities, as sequencing error may be falsely attributed to the presence of rare taxa and thus artificially inflate estimates of biological diversity [49]. However, the effects of different approaches for quality control of metabarcoded microbial community sequence data is insufficiently known. Bakker et al. [50] showed that different data processing pipelines can have serious effects on the interpretation of this type of data. When comparing the effects of algorithmic denoising (via Ampliconnoise) and a simple quality cleaning (quality filtering and similarity clustering), Bakker et al. [50] showed that across all samples Ampliconnoise recovered significantly less OTUs compared to the simple quality cleaning. They also showed that the number of sequences removed by Ampliconnoise was correlated with the diversity of the sample: this suggests that Ampliconnoise removes more sequences from more diverse samples. We emphasize that we still do not know whether pyrosequencing data cleaned via algorithmic denoising or simple quality filtering/similarity clustering reflects the true community structures better. There is a need for studies explicitly validating these methods using communities of known composition. When we performed a simple OTU picking of unpruned sequences, we recovered three times as many (621) clusters at 97% similarity, compared to the 179 clusters of pruned sequences. We consider that the application of algorithmic denoising via Ampliconnoise is the more conservative data pruning approach, but treating large datasets may be computationally difficult.

Two basic mechanisms possibly explain the patterns observed in the present study. First, host traits may promote infections with specific strains from the environment. Host morphology, physiology [15], microbial associations [16], [17], [51], host defense compounds [14], [17], [52], and genetic makeup [12], [18], [19] are all known to influence the taxonomic composition of fungal communities in plants. Host defense compounds may play an important role in structuring the fungal microbiome of balsam poplar. Balsam poplar excretes an odorous resin in the buds and leaves. It is possible that variances in quantity and chemical composition of the resin differs between host genotypes, and that affects fungal colonizations. The second mechanism at work might be vertical transmission. This implies that some of the observed fungi may overwinter in stem tissues or buds, and reinfect leaves every summer by growing into the leaves. Both scenarios suggest strong biological interactions between host and the foliar fungal microbiome.

Our results show that foliar fungal communities of trees growing in a common garden show host genotype-specific structures. This raises the possibility that at least some members of the leaf fungal community adapt to plant genotypes. Given that many endophytes alter plant fitness, this could be a form of coevolution. We hypothesize that host-foliar fungal relationships in trees might have been obscured by the low resolution of cloning and culturing methods available before next-generation metabarcoding. Most of the strongly discriminating taxa were relatively rare in the foliar fungal communities sequenced in this study (Table 1). Technological improvements in massively parallel sequencing considerably simplify the study of these diverse communities. Applying new methods for 454 sequence quality control and clustering showed that leaf-associated fungal communities may be less diverse than previously thought. Although these communities may still be considered very diverse, we think that proper application of methods in community ecology will help dealing with their complexity. We emphasize the importance of multivariate hypothesis testing in next-generation community analyses, instead of simple ordination tools for visual pattern discovery. Community ecology methods are highly suitable to test ecologically/evolutionarily important patterns in microbial community structure. The high resolution of next-generation metabarcoding opens new horizons in the study of host-fungal associations in trees. These tools may reveal previously overlooked tree-fungal relationships, especially if they are combined with genomic, molecular ecological and physiological data of the host tree.

## Supporting Information

Table S1 Collection data, 454 MID tags of the samples during sequencing, *a priori* LDA groups and LDA results of foliar fungal communities from balsam poplar.
(XLS)

Table S2 List and functions of foliar fungal taxa discriminating among host balsam poplar specimens on the basis of genotype group and regional translocation events.
(XLS)

Material S1 Pruned sequences produced by the 454 runs for the foliar fungal communities of each balsam poplar host specimen in FASTA format.
(ZIP)

Material S2 The number of pruned 454 reads assignable by MEGAN to low-level taxa (125), on the basis of BLAST hits against annotated fungal sequences present in GenBank.
(ZIP)

Material S3 Clusters delimited at sequence-divergence based equivalents of 6 grammar thresholds with GramCluster.
(ZIP)
